# Swithching Antipsychotic Medications in People with Schizophrenia: A 4-Year Naturalistic Study

**DOI:** 10.1192/j.eurpsy.2023.1335

**Published:** 2023-07-19

**Authors:** R. Ceres, M. Battipaglia, S. Donato, N. Attianese, G. M. Giordano, P. Bucci, G. Cascino

**Affiliations:** 1 University of Campania Luigi Vanvitelli, Naples; 2 University of Salerno, Scuola Medica Salernitana, Salerno, Italy

## Abstract

**Introduction:**

Although generally effective in ameliorating the core manifestations of schizophrenia, antipsychotics (APs) may lead to only suboptimal responses or may be associated with a variety of treatment-related adverse events which require additional treatment strategies. Under such clinical circumstances, switching APs represents a rational treatment option.

**Objectives:**

The present study aimed to identify the variables that predict AP switch and to quantify the frequency of this phenomenon in people with schizophrenia in real-life.

**Methods:**

A secondary analysis was conducted on the data collected at baseline and at a 4-year follow-up from a large sample of community-dwelling Italian people with schizophrenia. Demographic and clinical variables as well as information about AP treatment were recorded at two time points. Over the 4-year period, 34.9% of the 571 participants switched the AP; in particular, 8.4% of participants switched from first-generation APs (FGAs) to second-generation APs or vice versa, while 8.2% of them switched to clozapine.

**Results:**

Logistic regression models showed that combination of APs at baseline was negatively associated with AP switch, while treatment with FGAs and the presence of extrapyramidal symptoms at baseline were associated with AP class switch.

**Conclusions:**

Although the aim of the present study was not to assess predictors of clinical relapse in people with schizophrenia, we might speculate that switching APs represents a surrogate indicator of treatment failure in some patients and could lead into relapse, which is a costly aspect of schizophrenia management in both economic and human terms. The sooner such a negative outcome can be predicted and managed, the sooner the treatment can be optimized to avoid it.

**Disclosure of Interest:**

None Declared

